# Complete Genome Sequence of the *Blochmannia* Endosymbiont of *Camponotus nipponensis*

**DOI:** 10.1128/MRA.00703-20

**Published:** 2020-07-16

**Authors:** Jongsun Park, Hong Xi, Jonghyun Park, Sang June Nam, Yeong-Don Lee

**Affiliations:** a InfoBoss, Inc., Seoul, Republic of Korea; b InfoBoss Research Center, Seoul, Republic of Korea; c Research and Business Development Institute, Agricultural Corporation, Jeju Chunji, Republic of Korea; d Department of Agricultural Biotechnology, College of Agriculture and Life Sciences, Seoul National University, Seoul, Republic of Korea; e World Heritage Office, Jeju Island, Republic of Korea; Indiana University, Bloomington

## Abstract

*Blochmannia* endosymbionts, belonging to *Gammaproteobacteria*, live in bacteriocytes, which are specialized cells for these bacterial species in the *Camponotus* genus (carpenter ants). In this announcement, we describe the complete genome sequence of the *Blochmannia* endosymbiont of Camponotus nipponensis, which originated from a C. nipponensis colony collected in the Republic of Korea.

## ANNOUNCEMENT

Endosymbionts are very commonly identified among insect species (1). Ants of the genus *Camponotus* and its related genera have developed an essential bond with the gammaproteobacterial genus *Blochmannia*. They are found in bacteriocytes of the ants’ midgut, where they enrich the nourishment and immunity of the host ants (1–3). Genes for essential amino acid biosynthesis were retained during the reduction of the bacterial genome because they are crucial for nutritional transactions with the ants (4). Four complete genome sequences of these endosymbionts are now available (5–7), which can be fundamental resources for understanding the mode of action of interactions between ants and their symbionts.

Due to the rapid development of next-generation sequencing technologies, two whole and two mitochondrial genomes of the *Camponotus* genus are now available (8–10), demonstrating that genomic studies of this genus are becoming active and genomic data that can be utilized for identification of endosymbiont bacterial genomes using bioinformatic analyses are being accumulated. These data will also be useful for identifying the complete genomes of endosymbiotic bacterial species for an understanding of their coevolutionary histories. Here, we report the complete bacterial genome sequence of the *Blochmannia* endosymbiont isolated from Camponotus nipponensis.

A colony of Camponotus nipponensis was collected in the Gyorae Natural Recreation Forest on Jeju Island (33.441811N, 126.664388E), Republic of Korea (IN; Seoul, Republic of Korea; Jonghyun Park; voucher number KFDS00051). Its total DNA was extracted from multiple C. nipponensis workers by using a DNeasy blood and tissue kit (Qiagen, Hilden, Germany).

The sequencing library was constructed using the TruSeq Nano DNA library preparation kit (Illumina, San Diego, CA), following the manufacturer’s recommendations, with ∼350-bp DNA fragments. Genome sequencing was performed with the HiSeq X platform at Macrogen, Inc. (Republic of Korea), using extracted DNA from Camponotus nipponensis, which yielded 29.52 million 151-bp reads. Bioinformatic analyses were performed with default parameters except where otherwise noted. *De novo* assembly of bacterial genome sequences was done by Velvet v1.2.10 (11) with the option of a minimum depth of 50×, after filtering of raw reads using Trimmomatic v0.33 (12). Contig sequences obtained from Velvet assembly results were selected based on the results of a BLASTn search against the nonredundant data set, indicating that matched sequences were from endosymbiont bacteria (query coverage was >95%, and nucleotide identity was >80%). To complete the bacterial genome, SOAP GapCloser v1.12 (13), BWA v0.7.17 (14), SAMtools v1.9 (15), and Geneious R11 v11.1.5 (Biomatters Ltd., Auckland, New Zealand) were used to fill gaps and to conform extended sequences. The assembled genome sequence was confirmed as a circular genome via overlapped 5-kb sequences in both ends of the sequence. After completion of the bacterial genome sequence, all bases were confirmed using BWA v0.7.17 (14) and SAMtools v1.9 (15). Genome annotation was conducted using the RAST server (16).

The complete genome of the *Blochmannia* endosymbiont of Camponotus nipponensis was 788,522 bp long, with a GC content of 29.0%. It is the second longest genome of the *Blochmannia* endosymbiont, after that of “*Candidatus* Blochmannia pennsylvanicus” strain BPEN (GenBank accession number NC_007292.1) (5). This genome contains 631 protein-coding sequences, which is the same as the number for “*Candidatus* Blochmannia vafer” strain BVAF (CP002189.2) (6), the smallest number among the four *Blochmannia* genomes. Sequences of 40 tRNA genes, 1 5S rRNA gene, 1 16S rRNA gene, and 1 23S rRNA gene were also identified. The number of tRNAs found in our genome is the same as that of “*Candidatus* Blochmannia pennsylvanicus” strain BPEN (NC_007292.1) and is larger than those of the remaining three genomes. Forty of 631 protein-coding genes (6.33%) are for hypothetical proteins, which is much higher than the values for the four available genomes (ranging from 1 to 3 genes). We expect that our genome sequence will be used for comparative genomic analyses of this endosymbiont species to unravel the history of coevolution between *Blochmannia* endosymbionts and its host ants.

### Data availability.

The whole-genome project of the *Blochmannia* endosymbiont of Camponotus nipponensis has been deposited at DDBJ/ENA/GenBank under the accession number CP046534, BioProject number PRJNA592763, and BioSample number SAMN13439371. Raw sequencing data have been deposited under the accession number SRR10605340.
